# Docking and QSAR of Aminothioureas at the SARS-CoV-2 S-Protein–Human ACE2 Receptor Interface

**DOI:** 10.3390/molecules25204645

**Published:** 2020-10-12

**Authors:** Wojciech Płonka, Agata Paneth, Piotr Paneth

**Affiliations:** 1 Center for Bioinformatics (ZBH), Universität Hamburg, 20146 Hamburg, Germany; w.plonka@fqs.pl; 2 FQS-Fujitsu Poland, Parkowa 11, 33-332 Kraków, Poland; 3 Department of Organic Chemistry, Faculty of Pharmacy, Medical University of Lublin, Chodźki 4a, 20-093 Lublin, Poland; agata.paneth@umlub.pl; 4 Institute of Applied Radiation Chemistry, Faculty of Chemistry, Lodz University of Technology, Żeromskiego 116, 90-924 Lodz, Poland; 5 International Center for Research on Innovative Biobased Materials (ICRI-BioM)—International Research Agenda, Lodz University of Technology, Żeromskiego 116, 90-924 Lodz, Poland

**Keywords:** SARS-CoV-2, aminothioureas, docking, QSAR, ADMET

## Abstract

Docking of over 160 aminothiourea derivatives at the SARS-CoV-2 S-protein–human ACE2 receptor interface, whose structure became available recently, has been evaluated for its complex stabilizing potency and subsequently subjected to quantitative structure–activity relationship (QSAR) analysis. The structural variety of the studied compounds, that include 3 different forms of the N–N–C(S)–N skeleton and combinations of 13 different substituents alongside the extensive length of the interface, resulted in the failure of the QSAR analysis, since different molecules were binding to different parts of the interface. Subsequently, absorption, distribution, metabolism, and excretion (ADME) analysis on all studied compounds, followed by a toxicity analysis using statistical models for selected compounds, was carried out to evaluate their potential use as lead compounds for drug design. Combined, these studies highlighted two molecules among the studied compounds, i.e., 5-(pyrrol-2-yl)-2-(2-methoxyphenylamino)-1,3,4-thiadiazole and 1-(cyclopentanoyl)-4-(3-iodophenyl)-thiosemicarbazide, as the best candidates for the development of future drugs.

## 1. Introduction

With the outbreak of the severe acute respiratory syndrome coronavirus 2 (SARS-CoV-2) [1] devastating pandemic that has already claimed the lives of over one million people worldwide [2], therapeutics are urgently needed. For future prevention, a few vaccine strategies are being tested. At the same time, medicines that can be used in fighting the infection are being sought. It is thus not surprising that vigorous research is being carried out worldwide. One of the targets of both types of studies is the interface between the virus S-protein and the ACE2 receptor, focused on complex stabilizers and protein–protein binding inhibitors. Recent studies in this area include experimental [3,4] and theoretical [5,6,7,8] structural analyses, as well as mutagenic studies [9]. Advances from all these studies have been summarized [10].

The family of coronaviruses is relatively well known due to the previous severe human epidemics of SARS [11] and Middle East respiratory syndrome (MERS) [12]. In the search for medicines instantly available, repurposing of already approved drugs is studied as the first line of drug research. This approach has been attempted during the MERS epidemic [13] and is also being tried currently [14,15,16,17,18]. In the first of these studies, an NIH library of over 700 compounds was screened experimentally, identifying around 80 molecules with an anti-coronavirus effect. The most extensive study [19] concentrated on the interaction of the spike protein (S-protein) of the virus with the human ACE2 receptor, which is thought to be responsible for viral recognition by the host cells. By combining virtual high-throughput screening with ensemble docking of over 9000 compounds from the SWEETLAND library [20] and by using SARS-CoV-2 S-protein (NCBI: YP_009724390.1) and human ACE2 receptor (PDB: 2AJF) as templates to generate the SARS-CoV-2 S-protein–ACE2 receptor complex model, around 80 compounds already in clinical use that should exhibit anti-coronavirus activity have been identified. The S-protein–ACE2 receptor interface is continuing to be a subject of vigorous structural [3,4] and mutagenic [9] experimental studies, as well as of theoretical assessments [5,6,7,21]. The implications of these studies for therapy have been summarized recently [10].

While repurposing of compounds currently in medical use is necessary for a quick response to this public health threat, in the long run, drugs specific to this particular virus will be needed. With this aim in mind, we used the SARS-CoV-2 S-protein–ACE2 receptor complex model and its components to evaluate the binding properties of thiosemicarbazides, thiadiazoles, and triazoles that we previously studied for their anti-*Toxoplasma gondi* [22,23,24,25], antiviral [26], antibacterial [27,28,29,30,31,32], anticancer [17,33,34,35], anticonvulsant [36,37,38], analgesic [39,40] activities.

Docking studies allowed the comparison of the binding affinities of the considered compounds to the S-protein and ACE2 receptor interface. Subsequently, we carried out a quantitative structure–activity relationship (QSAR) analysis in the attempt to predict the binding properties of other compounds in the studied class. Unfortunately, the QSAR analysis failed to provide the expected guidance for this purpose. The probable reasons for this failure are discussed. We, therefore, carried out an absorption, distribution, metabolism, and excretion (ADMET) analysis (with a toxicity analysis restricted to the most promising lead candidates). Combined, our results point to two molecules among the studied compounds, i.e., 5-(pyrrol-2-yl)-2-(2-methoxyphenylamino)-1,3,4-thiadiazole and 1-(cyclopentanoyl)-4-(3-iodophenyl)-thiosemicarbazide, as the best candidates for the development of future drugs.

## 2. Results

**Figure 1:**
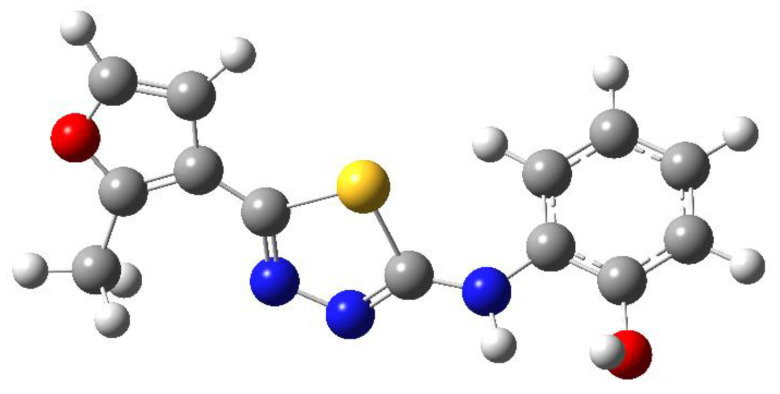
Chemical structure of the compound **FSoOH**.

The main focus of the present study was on the binding of selected molecules to the SARS-CoV-2 S-protein–human ACE2 receptor interface, although binding to the individual S-protein and ACE2 receptor was also analyzed. Three classes of compounds containing an N–N–C(S)–N skeleton, in a linear or cyclic topology, used in our laboratory in recent years for their inhibitory activity against several enzymes, were studied. Their structures contain three main cores: a linear carbonylthiosemicarbazide or its two cyclic derivatives, 1,3,4-thiadiazole and 1,2,4-triazole, each decorated with two substituents. As the C-substituent, one of the four five-member rings was used, while the N-substituent was a benzene ring or its *ortho*, *meta*, or *para* mono-substituted derivatives. These components are listed in Table 1, where also partial codes for the different moieties are provided (in bold). Thus, for example, the compound code **FSoOH** corresponds to 5-(2-methylfuran-3-yl)-2-(2-hydroxyphenylamino)-1,3,4-thiadiazole, illustrated in Figure 1 (this turned out to be the best ligand from among the studied compounds, as shown below). In total, 166 of the above compounds were considered.

The docking results for all compounds are available in the Supplementary Material for the interface between the S-protein of the virus and the human ACE2 receptor (as well for these two proteins separately). In Table 2, compounds with the best docking scores for both the interface and the individual proteins are listed. Note that algorithms implemented in docking programs use different mathematical formulas. In the case of the Gold program (and some other programs as well) the more favorable interactions the higher score. The values in bold indicate the best result for the interface (or each individual protein); the subsequent two values are written in italic. 

**Figure 2:**
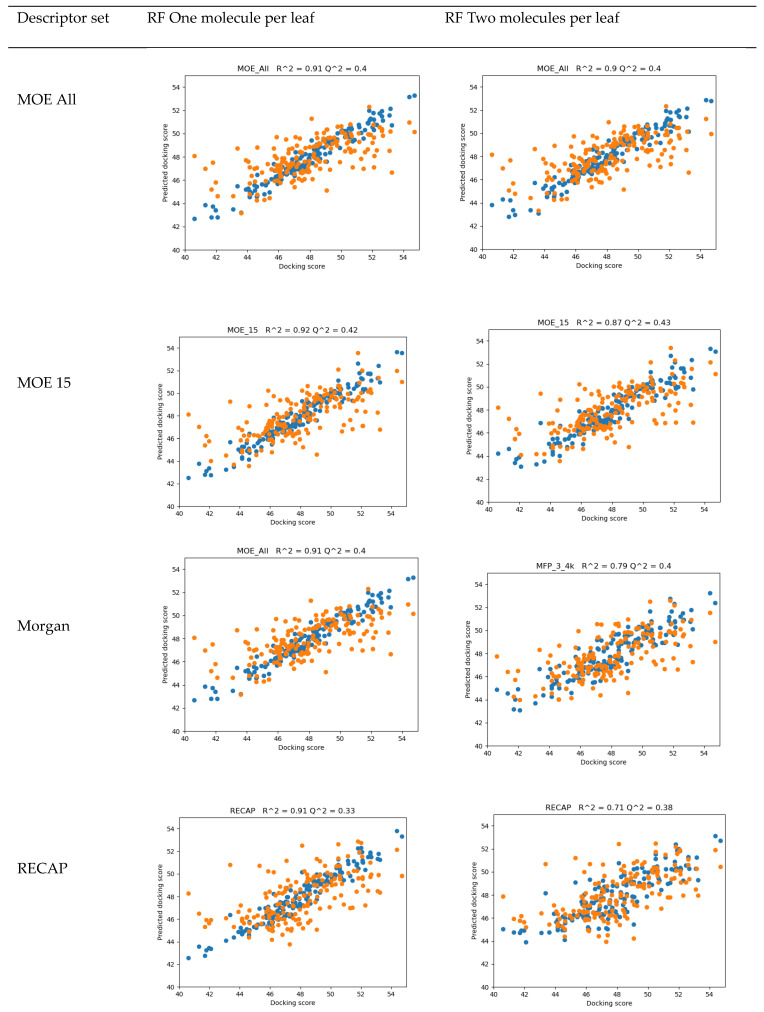
Results of the evaluation of Random Forest Regressor models on the training set (blue points) and leave-one-out cross-validation (orange points) for various considered descriptor sets.

**Figure 3:**
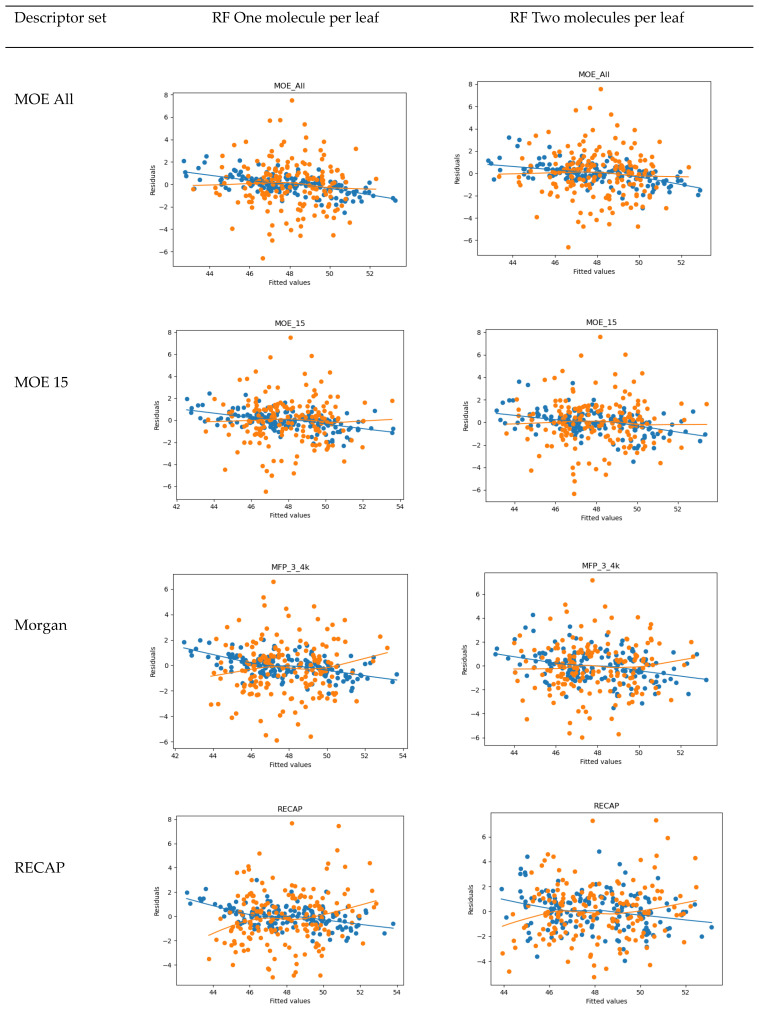
Diagnostic plots for the Random Forest Regressor models on the training set (blue points) and leave-one-out cross-validation (orange points) for various considered descriptor sets.

The QSAR results are shown in Figure 2. The best values of R^2 are in the order of 0.9; however, Q^2 values for the leave-one-out validation are 0.43 or lower. The analysis of residuals, presented in Figure 3, suggested that no particular molecule can be treated as an outlier. The Random Forest’s “min_samples_leaf” parameter controls whether decisions of a given tree can be made on the basis of a single sample (min_samples_leaf =1, the default) or of a specified number of samples. Increasing the min_samples_leaf usually lowers the fit of the model for the training set but improves predictive capabilities. Due to the small number of samples available, we investigated Random Forests with min_samples_leaf = 1 and 2 only.

## 3. Discussion

**Figure 4:**
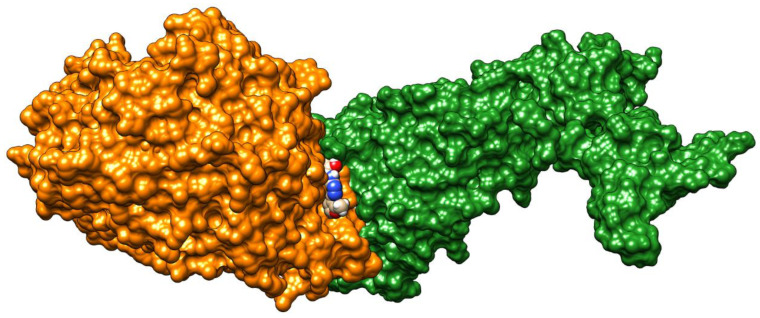
The best binding pose of **FSoOH** at the virus spike protein (S-protein, green)–human ACE2 receptor (orange) interface.

**Figure 5:**
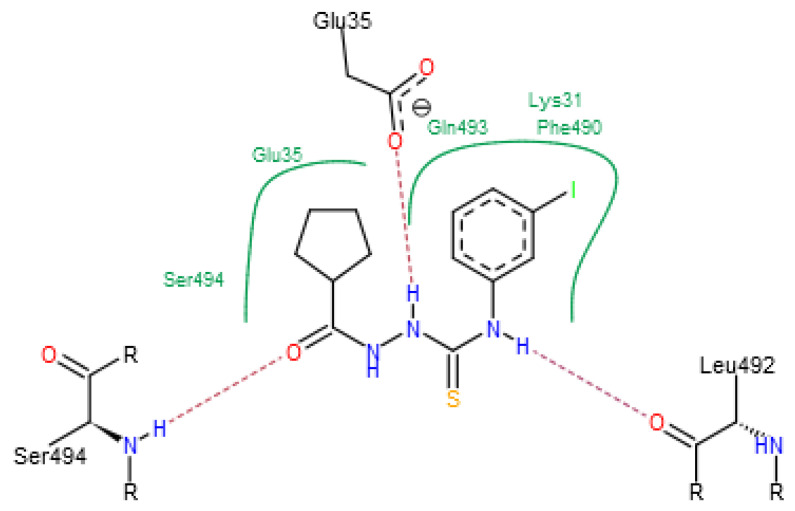
Interactions of **CCmI** in the binding groove of the interface.

As can be seen from the docking results presented in Table 2, **FSoOH** yielded the best results for the interface as well as for the human ACE2 receptor. The position of its best binding pose is illustrated in Figure 4. This molecule binds strongly to the Leu29–His34 fragment of the terminal helix of the ACE receptor and, to a lesser extent, to the Pro49–Tyr495 fragment of the S-protein. Among the N–N–C(S)–N motifs, the thiadiazole ring is the moiety most frequently present in top binding compounds. In this group, substituents attached to the nitrogen atom are most frequently substituted in the *ortho* position by fluorine or a hydroxyl group. Furan is the substituent of choice on the carbon side. It is worth noticing that the compound **CCmI** (together with **ICpNO_2_** and **CCpNO_2_**) was shown not to be cytotoxic in our recent studies on anti-*T. gondii* activity (unpublished results). For this ligand, the molecular interactions in the binding groove are illustrated in Figure 5.

In an attempt to extract the knowledge required for the design and synthesis of new compounds similar to those in our set but with improved activity, we used Machine Learning and performed a QSAR analysis of the S-protein–ACE2 interface docking scores. We wanted to investigate the relationships between activity and general physicochemical properties of the compounds, general structural features, as well as features specific to our molecules. We employed similar techniques used in the past [41,42], which proved successful. We chose the Random Forest Regressor as a modeling algorithm, due to its ability to handle non-linear relations and the possibility of learning from a small dataset with a large number of features.

It can be observed that Random Forests with one molecule per leaf described the training set better than those with two molecules per leaf. General physicochemical and topological descriptors (MOE All, MOE 15) worked better than fingerprints, while counts of fragments characteristic to the set performed poorly. This suggests that general shape-related and physicochemical properties of molecules are more important for activity than particular substituents present in our set of compounds. It should also be noted that there was almost no difference in the behavior of the models built with 354 MOE 2D descriptors and a limited set of 15, confirming the Random Forests’ capability to handle feature selection issues automatically. The leave-one-out cross-validation R^2 of about 0.4 of the models, however, did not encourage their specific use for judging the potency of new compounds; evidently, for this purpose, more training data are needed.

**Figure 6:**
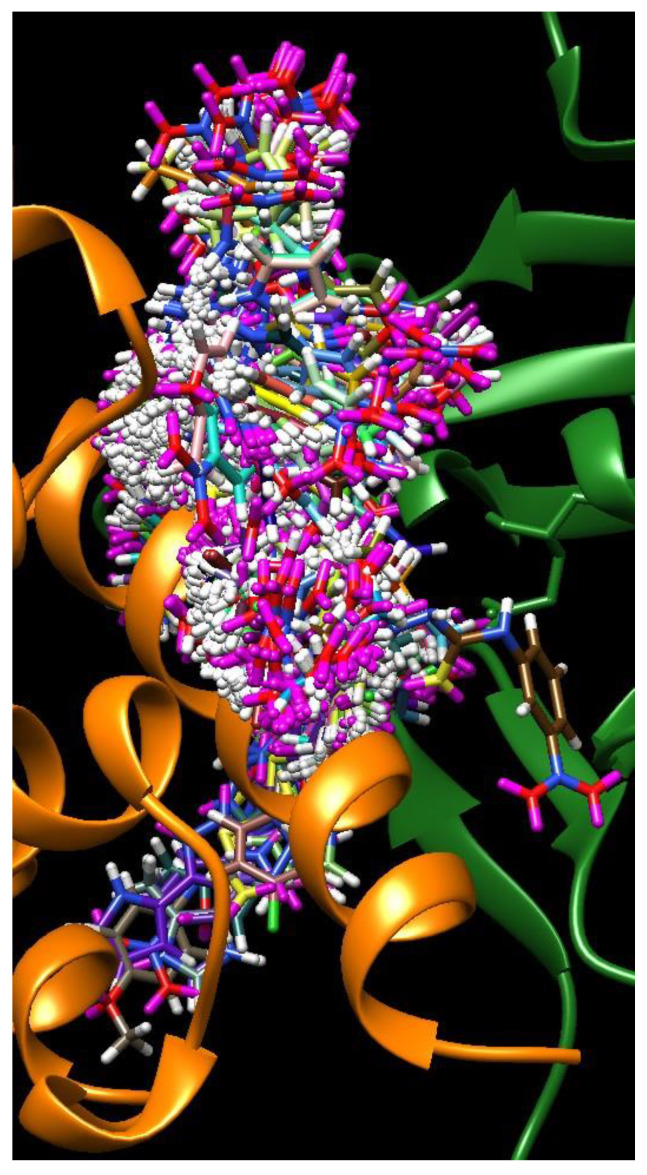
Overlay of the 10 best binding poses of all studied compounds in the groove of the S-protein–ACE2 receptor interface (for color code, see Figure 4).

This somewhat disappointing performance of the QSAR analysis may have several causes. Firstly, the number of studied molecules and their structural variety could have been too limited. However, since our previous experience with a similar technique using much smaller sets was quite positive [24,25] we think the most important reason was the size of the interface. This spans for nearly 45 Å in contrast to the quite confined size of a typical active site of an enzyme. This has several consequences. One of them is the fact that different molecules can bind to different parts of the interface groove, steered by forces (van der Waals, hydrogen bonding, electrostatic interactions, etc.) in different combinations. This is illustrated in Figure 6, which presents an overlay of the best 10 binding poses for each of the studied molecules.

**Figure 7:**
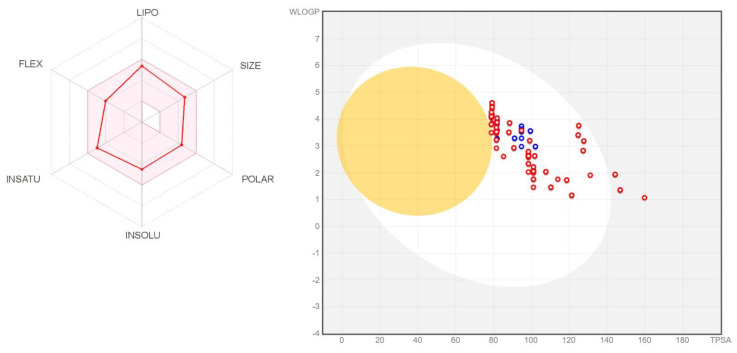
Results of the absorption, distribution, metabolism, and excretion (ADME) assessment. **Left** panel: consensus values for a single compound (**CCmI**). **Right** panel: BOILED-Egg graph [43] illustrating good gastrointestinal absorption (white area).

In the absence of clear indicative QSAR results that would allow us to scrutinize compounds outside the studied set, we analyzed the ADMET properties of the compounds used in the docking and QSAR studies, in search for the best lead candidates within this set. The ADME analysis comprised the evaluation of physicochemical parameters, druglikeness, lipophilicity, pharmacokinetics, and leadlikeness. A typical collection of consensus parameters for a given compound is illustrated in the left panel of Figure 7 for **CCmI**, while all individual values are presented in the Supporting Information (Table S5). The right panel of Figure 7 illustrates a comparison of the results obtained for all comperrorsounds. As can be seen, with a few exceptions, all points lie in the white area of the graph, relating logP to polar surface area (TPSA), indicating good oral bioavailability.

Since the ADME analysis also turned out not to be significantly discriminative for the studied compounds, we have carried out toxicity studies using statistical models. These calculations were performed only for the compounds listed in Table 2. The results of the tests are given in the last three columns of this table. The last column reports values relative to those obtained for chloroquine, a medicine considered for repurposed used against coronavirus disease 2019 (COVID-19), which, with the ChemPLP score of 66.29, exhibits strong complex-stabilization properties. As can be seen, toxicity results advocate against considering the compounds **PSoF** and **FCpF**, while favoring **PSoMe** and **CCmI** in further drug development studies.

## 4. Materials and Methods 

### 4.1. Docking

We used the published model [19] of the interface between the viral S-protein and the human ACE2 receptor that was constructed using SARS-CoV-2 S-protein (NCBI: YP_009724390.1) and human ACE2 receptor (PDB: 2AJF) as templates. The structure of this model has been recently confirmed experimentally [44]. A few docking algorithms have been tested. Initially, following the literature data, Vina [45] docking program was used. However, the scores obtained for the strongest inhibitors were very close and not discriminating. We, therefore, switched to the FlexX algorithm [46], as implemented in the LeadIT platform [47]. The scoring function of this program did a better job in the differentiation of the binding affinities of the studied compounds but showed some constraints, the most serious being the limit on the size of the acceptor, which precluded, in some cases, searches of the whole enzyme. SwissDock [48] in our hands also turned out not practical, as only a single ligand per submission to the server was possible. We therefore finally decided to continue with the ChemPLP algorithm [49] as implemented in the Gold program [50]. It is also noteworthy that this algorithm has been evaluated as one of the best in the most recent benchmark studies [51].

For the preparation and visualization of proteins and ligands, apart from those embedded in the docking programs, Hyperchem [52], Gaussview [53], Chimera [54], and Mercury [55] were used.

### 4.2. QSAR

Four sets of different molecular descriptors were used:

- All MOE [56] descriptors, as a measure of general properties, see Table S3
- 15 MOE 2D descriptors selected by Particle Swarm Optimization using Fujitsu ADMEWORKS ModelBuilder software [57] (‘“ASA_H”, “a_acc”, “a_nH”, “a_nN”, “E_ele”, “E_oop”, “GCUT_PEOE_3”, “GCUT_SLOGP_1”, “opr_violation”, “PEOE_VSA_PPOS”, “rsynth”, “SlogP_VSA6”, “SMR_VSA1”, “vsurf_CW5”, “vsurf_W5”), which were found to correlate best with activity in a linear combination, as a low-count set of general properties
- Morgan Fingerprints [58] with Radius = 3 and bit length of 4096 as general structural features
- Counts of fragments frequently appearing in our compound set obtained by the RECAP algorithm using Fujitsu ADMEWORKS ModelBuilder software, as features most specific to our set, see Table S4

Random Forest Regressor has been used as a modeling algorithm. The R^2 on the whole training set and R^2 of the leave-one-out cross-validation were used as metrics for learning and prediction performance, respectively. Calculations were done using Python scripts in the Anaconda environment [59] and the Fujitsu ADMEWORKS ModelBuilder software. Models were built using scikit-learn [60] of Random Forest Regressor with default parameters, except min_samples_leaf, as described in the Results section, and fixed random seed for reproducibility.

### 4.3. ADMET

The SwissADME program [61] implemented online [62] was used for the assessment of the ADME properties of all the studied compounds. The BOILED-Egg graph, which uses logP calculated by the Wildman and Crippen method [63] and TPSA [64], was generated at the same site.

Toxicology studies were carried out using online implementation [65] of the PreADMET program [66]. From among available statistical models, Ames TA100_10RLI/TA100_NA/ TA1535_10RLI/TA1535_NA tests on *Salmonella typhimurium* bacterial strain [67] and mouse [68] carcinogenicity, and *Daphnia* fish [69] toxicity were carried out. 

## 5. Conclusions

The presented docking studies identified 5-(2-methylfuran-3-yl)-2-(2-hydroxyphenylamino)-1,3,4-thiadiazole as the molecule with the best docking score among the studied set of nearly 170 compounds containing the –N–N–C(S)–N– motif. The resulting scores, when subjected to QSAR analysis did not, however, yield a model useful for a rational search of other related molecules. The most probable reason for this is the length of the interface binding groove, resulting in different binding forces depending on the exact site of the best interactions between proteins and ligands.

**Figure 8:**
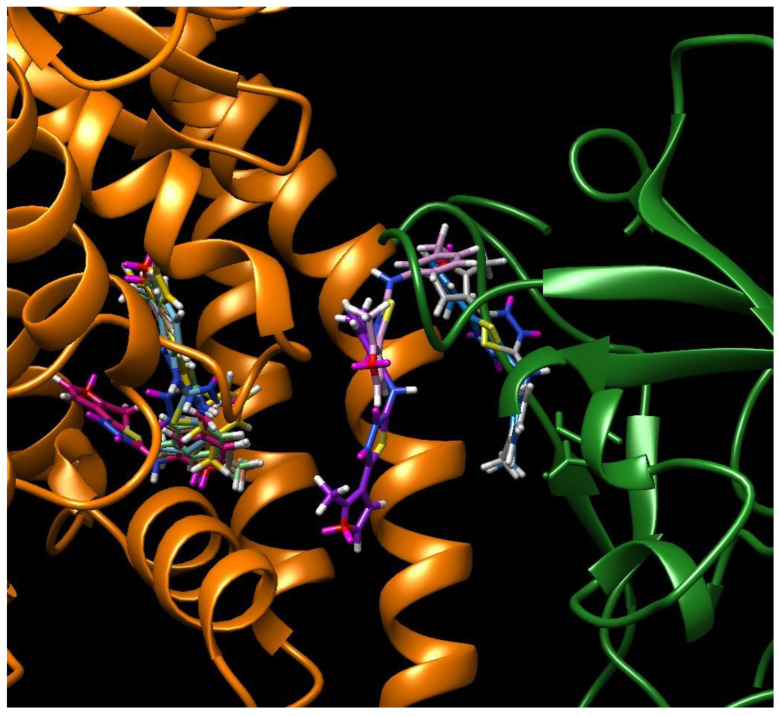
Overlay of the 10 best binding poses of **PSmMe** compound in the groove of the S-protein–ACE2 receptor interface (for color code see Figure 4).

As a side effect of our calculations, yet another warning for the type of studies presented here can be indicated. Attempts are being made to use massive docking analyses for large libraries of ligands in rational drug design (see for example, reference 11 for studies on the S-protein–ACE2 receptor interface.) These studies are excessively automated, leading to thousands of poses that can hardly be reviewed manually, as was done in this contribution. However, as depicted in Figure 8 for the compound **PSmMe**, the ligand can bind several neighboring sites. In fact, in this case, binding inside the ACE2 receptor (two poses on the left side) exhibited much higher scores (57.22 and 59.22) than binding at the S-protein–ACE2 receptor interface. This example illustrates possible pitfalls of automatic docking and advocates for a rectangular definition of the docking space (available, for example, in Vina) rather than for the more popular docking within a given radius from a molecule, residue, or point. 

Overall, our combined docking, QSAR, and ADMET studies led us to the conclusion that two of compounds among the studied aminothioureas, with high, although not the highest, docking scores are suitable for further search for a drug against COVID-19 due to their predicted low toxicity. These molecules are 5-(pyrrol-2-yl)-2-(2-methoxyphenylamino)-1,3,4-thiadiazole (**PSoMe**) and 1-(cyclopentanoyl)-4-(3-iodophenyl)-thiosemicarbazide (**CCmI**). The latter showed in the *Daphnia* toxicity test results even better than those of chloroquine, which is used in medical practice. We confirmed its low toxicity experimentally also in other studies.

## Figures and Tables

**Table 1:** Lists of structural fragments of the compounds used in the current study.

C-substituents R^1^	Core	N-substitutents R^2^
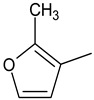 2-methylfuran-3-yl (**F**) 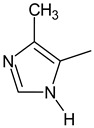 4-methyl-imidazol-5-yl (**I**) 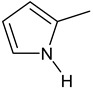 pyrrol-2-yl (**P**) 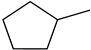 cyclopentyl (**C**)	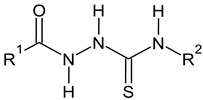 carbonylthiosemicarbazide (**C**) 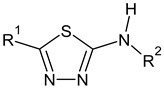 1,3,4-thiadiazole (**S**) 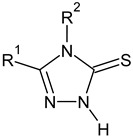 1,2,4-triazole-3-thione (**T**)	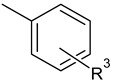 R^3^ = **H**, *ortho*, *meta*, or *para*: **F**, **Cl**, **Br**, **I**, **OH**, **OMe**, **Me**, **NO_2_**

**Table 2:** ChemPLP docking scores of the studied compounds in relation to S-protein, ACE2 receptor, and their interface and toxicity results for statistical bacteria, mammals, and fish models.

Compound	Interface	Virus S-Protein	Human ACE2 Receptor	TA100/TA1535 Ames Tests	Carcino (Mouse) Test	*Daphnia* Test (vs. Chloroquine)
**FSoOH**	**54.71**	39.89	**53.16**	−+++	negative	3.0
**PSoF**	*53.96*	*49.01*	47.68	all positive	positive	1.8
**FCmOH**	*53.67*	46.07	*51.68*	−+++	negative	3.3
**PSoMe**	52.09	**49.05**	47.45	+−−+	negative	1.3
**CCmI**	53.25	41.24	*52.6*	+−++	negative	0.9
**FCpF**	46.48	*49.01*	45.56	all positive	positive	2.5

## Supplementary Materials

The following are available online, Docking scores obtained for all studied compounds, MOE descriptors, list of substructures generated by the RECAP algorithm used as counts in QSAR modeling, and results of ADME calculations can be found at https://doi.org/10.6084/m9.figshare.12130932.v3.
